# Dallas Clinics

**Published:** 1915-12

**Authors:** 


### Dallas Clinics.

Among the interesting cases reported at the clinics held in Dallas as part of the meeting of the Southern Medical Association were two presented by Dr. C. M. Rosser and Dr. J. B. Smoot. The case presented by Dr. Rosser was that of Ernest Shokley of Ore City,

Texas, and was perhaps the most interesting of any of the clinics. Twenty years ago Shokley was injured in the cyclone at Sherman. For several years after the cyclone there was apparently no evil effects from the injuries received by Shokley at the time.

After a few years, however, Shokley began to suffer with severe headaches and often with convulsions. Seven years ago he was brought to the Baptist Sanitarium and Dr. Rosser, who was then professor of surgery at the sanitarium, operated on him. He removed the depressed part of the skull and a tumor which had formed and was pressing on the young man's brain. Dr. Rosser then removed one of Shokley's ribs and taking a piece three and one-half inches in length he replaced with it the part of skull which had been removed.

At the present time this piece of rib is now solidly grown together with the balance of the skull and Shokley, to all appearances, will never again be bothered with any trouble as a result of the old injury.

The case presented by Dr. Smoot and which aroused much interest among the medical men was the grafting of pig skin onto a large section of burned flesh. This operation was performed some time ago on a young girl at the city hospital. The skin of the young pig which was granted onto the burned places is now beginning to grow and it is believed that the operation will prove to be highly successful and of great benefit to the patient.

Clinics are being held Saturday's at the various hospitals and a number of other interesting cases are being cited by local doctors for the benefit of the visiting brethren.

Dr. S. E. Milliken, professor of surgery and a post-graduate of Polyclinic, held an unusually interesting clinic in tendon grafting and other methods used in club-foot deformity.

Dr. J H. Smart, professor of surgery in the same institution, performed several operations, among which was a double pyosalphinx and the hysterectomy.

Dr. H. M. Doolittle performed an abdominal operation and exhibited two cases of congenital pyloric stenosis, posterior gastroenterostomy, pernicious anemia, gall bladder and Pott's disease.

Dr. C. M. Rosser operated for a tumor of the breast and did a resection of a portion of the lower one-third left ulna bone to correct a deformity of the left wrist. He presented a goiter case.

From the time Dr. Worley placed the mask over the face of the patient who was operated on for breast tumor but one minute and fifteen seconds passed before Dr. Rosser was operating. In placing the patient under the nitrous-oxide anesthesia, Dr. Worley called attention to the fact that with the use of nitrous-oxide the patient was asleep in fifteen seconds, the usual time required. The patient wakened in twenty seconds after the mask was removed from the face. In administering this form of anesthesia all patients are given a hypodermic of one-sixth grain of morphine, one one hundred and fiftieth grain of acopolomine, one hour before the operation. Twenty grains of chloretone is also given preparatory to the operation to combat nausea or vomiting.

Nitrous-oxide was used in operations by Dr. C. M. Rosser, Dr. Elbert Dunlap and Dr. Edward H. Cary.

### TWO OPERATIONS ON PATIENT.

Dr. Hal Gambrell performed two operations on one patient—one for perineorrhaphy and the other for gall bladder. Dr. Cary operated for cataracts, mastoids and glaucoma. Dr. H. H. Martin, of Savannah, Ga., operated for Gasserian ganglion. Dr. Elbert Dunlap operated at the clinic building for double salpingo-oophorectomy. A study of the gastro-intestinal tract was presented by Dr. J. M. Martin through the use of the X-ray machine.

Dr. J. B. Shelmire and Dr. W. J. Calvert presented several cases. Dr. C. M. Grigsby showed two cases of mitral disease, one aortic aneurism, one aortic regurgitation, one nephritic heart and one arterio-sclerotic heart.

